# Gene therapy of ovarian cancer using IL-21-secreting human umbilical cord mesenchymal stem cells in nude mice

**DOI:** 10.1186/1757-2215-7-8

**Published:** 2014-01-20

**Authors:** Yunxia Zhang, Jing Wang, Mulan Ren, Miao Li, Dengyu Chen, Junsong Chen, Fangfang Shi, Xiaoying Wang, Jun Dou

**Affiliations:** 1 Department of Gynecology & Obstetrics, Zhongda Hospital, Medical School, Southeast University, Nanjing 210009, China; 2 Department of Pathogenic Biology and Immunology of Medical College, Southeast University, Nanjing 210009, China; 3 Department of Oncology, Zhongda Hospital, Southeast University, Nanjing 210009, China

**Keywords:** Umbilical cord mesenchymal stem cells, Interleukin-21, Ovarian cancer, Gene therapy

## Abstract

**Background:**

The human umbilical cord mesenchymal stem cells (hUCMSCs) have the ability to migrate into tumors and therefore have been considered as an alternative source of mesenchymal progenitors for the therapy of malignant diseases. The present study was aimed to investigate effect of hUCMSCs as vehicles for a constant source of transgenic interleukin-21 (IL-21) on ovarian cancer in vivo.

**Methods:**

The hUCMSCs were engineered to express IL-21 via lentiviral vector- designated ‘hUCMSCs-LV-IL-21’, and then were transplanted into SKOV3 ovarian cancer xenograft-bearing nude mice. The therapeutic efficacy and mechanisms of this procedure on ovarian cancer was evaluated.

**Results:**

The isolated hUCMSCs were induced to differentiate efficiently into osteoblast and adipocyte lineages in vitro. The expressed IL-21 in the supernatant from hUCMSCs-LV-IL-21 obviously stimulated splenocyte’s proliferation. The hUCMSCs-LV-IL-21 significantly reduced SKOV3 ovarian cancer burden in mice indicated by tumor sizes compared with control mice. The expressed IL-21 not only regulated the levels of IFN-γ and TNF-α in the mouse serum but also increased the expression of NKG2D and MIC A molecules in the tumor tissues. The down regulation of β-catenin and cyclin-D1 in the tumor tissues may refer to the inhibition of SKOV3 ovarian cancer growth in mice. In addition, hUCMSCs did not form gross or histological teratomas up to 60 days posttransplantation in murine lung, liver, stomach and spleen.

**Conclusion:**

These results clearly indicate a safety and usability of hUCMSCs-LV- IL-21 in ovarian cancer gene therapy, suggesting the strategy may be a promising new method for clinical treatment of ovarian cancer.

## Background

More than forty years ago, the study showed that cells isolated from the stroma of bone marrow were capable of osteogenesis, suggesting the characteristics of mesenchymal stem cells (MSCs) [1]. Subsequent studies have demonstrated that MSCs exist not only in the bone marrow, but also in cord blood, cancellous bone, adipose tissue, synovium and umbilical cord [2-7]. The human umbilical cord contains an inexhaustible, noncontroversial source of stem cells [8]. Human umbilical cord mesenchymal stem cells (hUCMSCs) grow as adherent cells with mesenchymal morphology, have self-renewing, express cell surface markers displayed by MSCs, and may be differentiated into bone, cartilage, adipose, muscle, and neural cells, etc. The properties promotes their potential utility in several intractable human disease such as neurodegenerative disease, cancer, heart disease, etc. Since hUCMSCs have the ability to migrate into tumors, they could be used as vector for targeted cancer therapy [9,10].

There is increasing evidence that ovarian cancer is the first leading cause of death among gynecologic malignancies. The high mortality rate of ovarian cancer is usually attributed to late diagnosis of this tumor due to a lack of early symptoms. Even though the treatment of optimal cytoreductive surgery followed by systemic chemotherapy is initially effective in 80% of patients, recurrent cancer is inevitable in the vast majority of cases. Furthermore, recurred tumors became unresponsive to the addition of chemoradiation following an initial response [11,12]. The second-and third-line (and beyond) therapies are aimed at palliative care to prolong the time of tumor progression and to improve the patient’s life quality [13]. Therefore, the novel therapeutic strategies are desperately needed for treatment of this malignant tumor [11].

In our previous studies, interleukin-21 (IL-21) was involved in T and NK cell activation and induced a strong cell-mediated immune responses in the tumor vaccine approaches [14,15]. In fact, IL-21 has been extensively applied to significantly augment antitumor immunity in multiple murine tumor models and clinical trials, such as metastatic lymphoma [16], melanoma [17,18], ovarian cancer [19], etc. Previously, we constructed recombinant pIRES2-IL-21-EGFP and transfected it into CD34^+^ human umbilical cord blood stem cells (UCBSCs) for treatment of ovarian cancer. The animal experiment results indicated that transferring CD34^+^ UCBSC- IL-21 into A2780 ovarian cancer xenograft-bearing nude mice augmented the therapeutic effect on ovarian cancer, however, IL-21 expression was gradually decreased in the mouse tumor sites 21 days posttransplantation [20]. In order to last the expression of IL-21 in tumor-bearing mice, we used lentiviral vector as delivery of IL-21 in this study. Accumulating evidence indicates that lentiviral vector has evolved as a benchmark tool for stable gene transfer into cells with a high replicative potential and provides a rich resource for numerous applications in experimental platforms and therapeutic settings.

Since hUCMSCs have attracted much attention due to their availability, low immunogenicity, as well as strong tropism for tumors in contrast to hMSCs from other sources [8-10], we particularly focused on the investigation using hUCMSCs to express IL-21 via lentiviral vector to assess the therapeutic effect of hUCMSCs- LV-IL-21 on SKOV3 ovarian cancer xenograft-bearing mouse model in the current study. Here, we show that hUCMSCs themselves do not form tumors, and that tumor growth in ovarian cancer bearing mice is significantly inhibited following systemic administration of IL-21-expressing hUCMSCs.

## Methods

### Cell lines, mice, and primers

Human ovarian cancer cell line SKOV-3 and human embryonic kidney (HEK) 293T cells were purchased from the Cellular Institute in Shanghai, China. SKOV-3 cells were maintained in complete media consisting of RPMI 1640, 2 mmol/L-glutamine, 100 u/ml penicillin, 100 μg/ml streptomycin, and 10% fetal bovine serum (FBS, Gibco BRL, USA). HEK293T cells were cultured in DMEM medium plus 10% FBS, 2 mmol/LL-glutamine, 100 u/ml penicillin, and 100 μg/ml streptomycin. All cells were cultured at 37°C in a humidified 5% CO_2_ atmosphere.

Balb/c nude mice of 4–5 weeks of age were acquired from the Animal Center of Yang Zhou University of China and were raised under sterile conditions in air-filtered containers at the Experimental Animal Center, Medical School of Southeast University. All the experiments were performed in compliance with the guidelines of the Animal Research Ethics Board of Southeast University, China.

The used primer sequences are as follows:

The PCR primer sequence for IL-21 gene was 5′-GGCGCTAGCATGGATCG CCTCCTGATTAGACTT-3′ (sense), and 5′-TGCGCGGCGTCGGTGGAGAGAT GCTGAT-3′ (antisense). The PCR primer for natural killer glucoprotein 2 domain (NKG2D) gene was 5′-CTCCATTTTTTTTCTGCTGCTTC-3′ (sense), and 5′-AAG GCTGGCATTTTGAGACATAC-3′ (antisense). The PCR primer for MICA gene was 5′-AAGACCAAGACACACTATCACGC-3′ (sense), and 5′-GGTGTCGTGGC TCAAAGATAC-3′ (antisense). The PCR primer for actin gene was 5′-GTCCACCG CAAATGCTTCTA-3′(sense), and 5′-TGCTGTCACCTTCACCGTTC-3′ (antisense). All the primers were synthesized by Company of Gene and Technology of China in Shanghai.

### Isolation and culture of hUCMSCs

All human umbilical cord blood samples were obtained from the Department of Gynecology & Obstetrics of Zhongda Hospital, Southeast University (Nanjing, Jiangsu, China) and were used in accordance with the ethical guidelines and accepted human studies protocols at Southeast University School of Medicine. The length of the umbilical cords in pregnancy (more than 37 weeks) was about 15 cm. hUCMSCs were harvested as described previously [21,22]. To ensure a homogeneous population of stem cells, passage-4 cells were characterized by flow cytometry (FCM) to analyze specific surface antigens of MSC lineage including CD29, CD34, CD45, and CD90. Approximately 10^6^ cells per vial were used for each antigen detection. Nonspecific binding was first blocked with a staining buffer containing 2% FBS for 15 min, and cells were then incubated with single label antigen for 20 min on ice. Mouse isotype antigen served as a negative control. FCM analysis was conducted using a FAC scan flow cytometer (BD Biosciences). Passage-4 hUCMSCs were used to test the ability of osteoblastic and adipogenic differentiation as described in a previous reports [23-25].

### Construction of recombinant lentiviral vector containing IL-21 gene

To generate the IL-21 expression lentivirus vector, we amplified IL-21 by PCR from template plasmid pRSC-IL-21 using the designed primers as aforesaid. The lentivirus IL-21 and the lentiviral without IL-21 gene were produced from the transient transfection of the HEK293T cells with pHAGE-CMV-IL-21-IZsGreen, psPAX2, and pMD2.G plasmid DNAs (kindly provided by Professor Lu Chun, Department of Microbiology and Immunology, Nanjing Medical University, China) plus Lipofectamine 2000 (Invitrogen, USA) according to the manufacturer’s protocol [26]. The IZsGreen expressed by HEK 293T cells was detected using fluorescence microscopy.

### Transduction and analysis of IL-21 expression

hUCMSCs were plated in 100 mm plates at 6 × 10^6^ cells per plate. After one day, the cells were 70% confluent. The cells were incubated sequentially with the 48 hour and 72 hour lentivirus-IL-21 and lentivirus supernatants respectively for 12 h. Following the last transduction, the cells were washed and incubated with fresh growth medium to allow puromycin-resistance expression. Two days later, puromycin selection was performed by incubating the cells in growth media supplemented with 1 μg/mL puromycin (Clontech Laboratories Inc.) for five days. After selection, hUCMSCs containing lentivirus-IL-21 (named hUCMSCs-LV-IL-21) and hUCMSCs lentivirus without IL-21 (named hUCMSCs-LV-Vec) were selected respectively. Then IL-21 expression was analyzed by western blotting. To identify the bioactivity of UCMSCs-LV-IL-21, the splenocytes from the normal nude mice were incubated with the supernatant from the cultured hUCMSCs-LV-IL-21 for 72 h, then the splenocyte proliferative activity was detected by FCM as described in a previous report [27].

### Evaluation of gene therapy of ovarian cancer efficacy

Balb/c nude mice of 4–5 weeks of age were randomly assigned to PBS control group, hUCMSCs group, hUCMSCs-LV-Vec group, and hUCMSCs-LV-IL-21. In establishing an ovarian cancer xenograft-bearing nude mice model, 5 × 10^6^ SKOV3 cells were subcutaneously injected into a mouse’s right flank. About 7 days after the injection, tumors were visible to the naked eye. Then, 100 μl PBS, 1 × 10^6^ hUCMSCs or hUCMSCs-LV-Vec or hUCMSCs-LV-IL-21 were injected into the mouse’s tumor sites, and tumor growth was monitored each day. The tumor volumes were evaluated every 3 days by measuring two perpendicular diameters of the tumors using calipers [28]. Three mice per group were used and the experiment was repeated two times. All mice were sacrificed at 35 days after injection of different hUCMSCs. In addition, 1 × 10^6^ hUCMSCs were injected into 5 nude mice to observe if the cells could form tumors by 60 days of observation.

### ELISA for IL-21, IFN-γ and TNF-α

Mouse serum cytokines were measured using a commercially available enzyme-linked immunosorbent assay (ELISA) according to the manufacturer’s protocol (eBioscience Company, USA). Briefly, samples were diluted 1:5 with PBS, and each cytokine was captured by the specific primary monoclonal antibody and detected by biotin labeled secondary antibody. The plates were read at 450 nm using a microplate reader (Bio-Rad Labs, Hercules, CA). The samples and standards were run in triplicate, and the sensitivity of the assays was 0.1 U/ml for interferon-γ (IFN-γ), IL-21, and tumor necrosis factor-α (TNF-α), respectively [29,30].

### RNA isolation and quantitative RT-PCR

Total RNA was obtained using the Qiagen RNeasy kit (Qiagen, CA). Isolated RNA samples were dissolved in RNase-free water, and RNA quantity was measured using NanoDrop (Thermo Fisher Scientific). cDNA was synthesised from 10 μg of total RNA at a volume of 100 μl using ImProm RT-II™ (Promega) according to the manufacturer’s instructions. cDNA samples were diluted with sterile deionised water to a total volume of 100 μl, and 2 μl was added to a PCR reaction. Quantitative RT-PCR (qRT-PCR) was performed on an ABI step one plus real-time system (Applied Biosystems, USA). We analyzed the relative expression levels of NKG2D and MIC A genes using the primers described above [31].

### Western blotting

The protocol was based on a previously published report [32]. Briefly, 1 × 10^6^ cells were collected and lyzed in a protein extraction buffer (Novagen, WI) according to the manufacturer’s protocol. Western blotting was performed after 12% sodium dodecyl sulfate-polyacrylamide gel electrophoresis and proteins (15 μg/lane) were electrotransferred onto a nitrocellulose membrane. The rabbit antibody specific to mouse IL-21 or human β-catenin or human cyclin-D1 was added to the membrane for 1 h, the membrane was washed for 5 min with antibody wash solution three times, and the subsequent steps were performed according to the Western-Breeze Kit’s protocol (Invitrogen). Immunoreactive bands were detected by the Odyssey scanning instrument (LICOR Odyssey, USA).

### Histopathology of tissues and tumors

Mice were sacrificed 35 days (gene therapy of ovarian cancer experiment) after the SKOV3 cell challenge or 60 days (tumor formation experiment) after the injection of hUCMSCs. The tissues of the spleens, livers, stomachs, and lungs as well as the tumor tissues were removed from the nude mice and fixed in 10% formalin, and then embedded in paraffin. Serial thin tissues and tumor sections (4 μm) were cut and mounted on SuperFrost Plus glass slides, fixed in methanol, and stained in hematoxylin and eosin (H&E) [33].

### Immunohistochemistry

4 μm formalin-fixed, paraffin embedded slides were incubated with the rabbit anti human β-catenin antibody or rabbit anti human cyclin-D1 antibody (Sigma) after over night incubation at 4°C. The samples were then labeled with horseradish peroxidase conjugated streptavidin (Invitrogen) and the chromogenic reaction developed using liquid DAB substrate pack (Biogenex, San Ramon, CA) according to the manufacturer’s instructions. β-catenin and cyclin-D1 stained cells from random and non-overlapping fields were counted under a light microscope, magnification of 200× [31].

### Statistical analysis

Statistical comparisons were performed using the Student’s *t*-test method or single factor analysis of variance to test for any statistically significant differences in the results between the experiment group and the control group. *P* < 0.05 was considered statistically significant. Analyses were performed with the Graph Pad Prism 5.0 statistical software package (Graph Pad Company, USA) [19].

## Results

### Culture and identification of hUCMSCs

**Figure 1:**
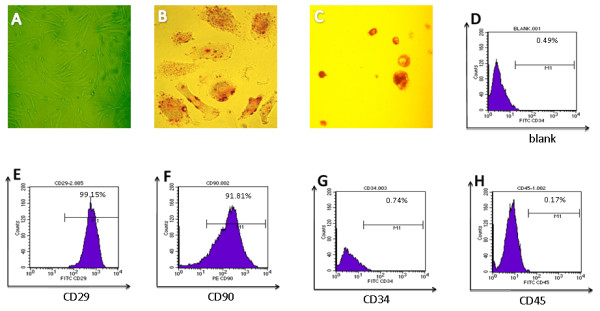
**Isolation and characteristic identification of hUCMSCs.** Panel **(A)** shows morphology of hUCMSCs at magnification 100×; Panels **B**-**C** exhibit that hUCMSCs were induced to differentiate into osteoblast **(B)** and adipogenesis **(C)** by osteogenic and adipogenic differentiation assays in vitro. Original magnification is 400×; Panels **(D-H)** indicate that the cultured Passage-4 hUCMSCs were phenotyped with different fluorescein isothiocyanate-labeled antibodies.

The photo of hUCMSCs shows that the cells formed whirlpool-like arrays and developed a defined spindleshaped fibroblastic morphology when a confluent monolayer (Figure 1A). The hUCMSCs in specific culture medium were observed to differentiate efficiently into osteoblast (Figure 1B) and adipocyte lineages (Figure 1C). These cells showed good homogeneity and expressed hUCMSC phenotypic characteristic markers such as positive phenotypes of CD29, and CD90 and negative phenotypes of CD34 and CD45 (Figure 1D-H). The data indicated that hUCMSCs were developed appropriately, which assists in the further study of hUCMSCs as vehicles for IL-21 delivery via lentiviral to develop therapeutic effect on SKOV3 ovarian cancer xenograft-bearing nude mice.

### Transduction and analysis of IL-21 expression

**Figure 2:**
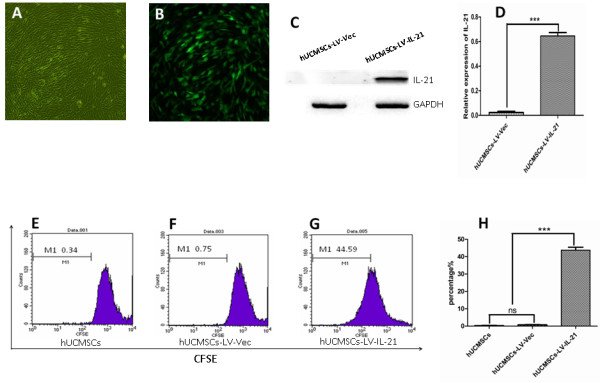
**Detection of IL-21 expression and its bioactivity in hUCMSCs transducted with recombinant lentiviral-IL-21.** Panels **A-B** indicate morphology of hUCMSCs transducted with recombinant lentiviral-IL-21 under a light microscope **(A)** and a fluorescence microscope **(B)** at magnification 100×; **C-D**. IL-21 expression was identified by Western blotting **(C)** and relative expression of IL-21 **(D)**; **E**-**G**. The effects of the supernatants from the cultured control hUCMSCs **(E)**, hUCMSCs-LV-Vec **(F)**, and hUCMSCs-LV-IL-21 **(G)** on the splenocyte’s proliferative activity, respectively; **H**. The statistical analysis of splenocyte’s proliferative activity. ***p < 0.005.

Construction of recombinant lentiviral containing IL-21 gene was confirmed by gene sequencing. pHAGE-IL-21, pMD2.G and psPAX2 were used to co-transfect the HEK293T cells, and the IZsGreen expressed by HEK 293T cells was observed under a fluorescence microscopy (These data not shown). Next, we developed the hUCMSCs-LV-IL-21 that were transducted with the lentiviral-IL-21. Three days post transduction, the IZsGreen expressed by hUCMSCs-LV-IL-21 cells was observed under a light microscope (Figure 2A) and a fluorescence microscopy (Figure 2B), respectively. The IL-21 expression in hUCMSCs-LV-IL-21 was analyzed by western blotting as is shown in Figure 2C. Whereafter, we further identified the bioactivity of IL-21 expressed by hUCMSCs-LV-IL-21. The isolated splenocytes from the normal nude mice were incubated with the supernatant from the cultured hUCMSCs- LV-IL-21 for 72 h. Figure 2E-H indicate the proliferative activity of splenocytes was significantly increased in the hUCMSCs-LV-IL-21 group (44.59%, G) compared with the control group (0.34%, E) and the hUCMSCs-LV-Vec group (0.75%, F), respectively (*P* <0.005). This result suggested that the IL-21 in the supernatant from cultured hUCMSCs-LV-IL-21 was functional, and that hUCMSCs would provide good vehicle for further hUCMSCs-based IL-21 gene therapy of ovarian cancer.

### Efficacy of hUCMSCs-LV-IL-21 therapy for ovarian cancer in nude mice and the related mechanism investigation

**Figure 3:**
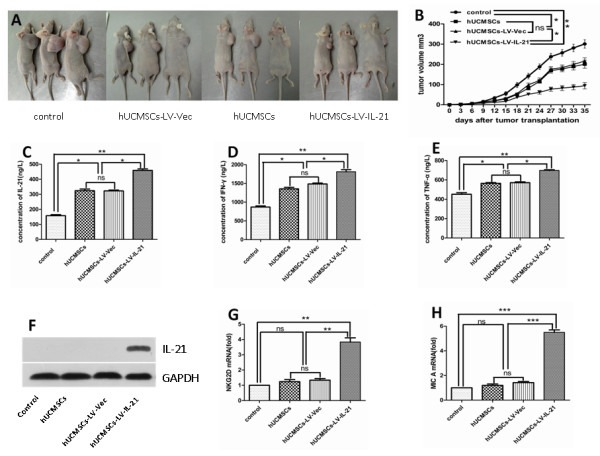
**Inhibition of ovarian cancer growth in mice treated with hUCMSCs-LV-IL-21. A**. The pictures represent the ovarian cancer bearing mice at five weeks after challenge of 5 × 10^6^ SKOV3 cells along with injection of various hUCMSCs; **B**. The tumor sizes were analyzed and indicated the 301.03 ± 23.78 mm^3^, 219.80 ± 15.67 mm^3^, 200.96 ± 19.97 mm^3^ and 94.20 ± 15.57 mm^3^ in the mice treated with PBS control, the hUCMSCs, hUCMSCs-LV-Vec and in the hUCMSCs-LV-IL-21 in order; **C**-**E**. Levels of IL-21, IFN-γ and TNF-α in murine serum were detected by ELISA assay, respectively; **F**. IL-21 expression was shown in the tumor tissues from the mice treated with hUCMSCs-LV-IL-21. However, no specific IL-21 band was shown in the tumor tissues from the mice treated with hUCMSCs-LV-Vec, hUCMSCs and PBS, respectively; **G**-**H**. The mRNA expression of NKG2D and MIC A detected by the real time RT-PCR in tumor tissues from the mice treated with hUCMSCs-LV-IL-21, hUCMSCs-LV-Vec, hUCMSCs and PBS, respectively. *p < 0.05, **p < 0.01, and ***p < 0.005. ns: no statistical significant.

In order to evaluate the efficacy of hUCMSCs-LV-IL-21 therapy for ovarian cancer in the mice, we first established the ovarian cancer bearing mouse model by injection of 5 × 10^6^ SKOV3 cells in a mouse’s flank, and then we tested whether injection of 1 × 10^6^ hUCMSCs-LV-IL-21 could inhibit the SKOV3 ovarian cancer growth in the mouse model. Figure 3A shows that hUCMSCs-LV-IL-21 obviously inhibited tumor growth compared with control mice. There were significantly decreased of tumor sizes in the hUCMSCs-LV-IL-21 group compared with the control group (*p* < 0.01), hUCMSCs group (*p* < 0.05) and hUCMSCs-LV-Vec group (*p* < 0.05), respectively, but there was no significant difference in tumor sizes between hUCMSCs group and hUCMSCs-LV-Vec group (*p* > 0.05). To further analyze the mechanisms of hUCMSCs- LV-IL-21 therapy for ovarian cancer in mice, we designed an experiment to find out whether the serum cytokines of IL-21, IFN-γ, and TNF-α were changed in the mice treated with hUCMSCs-LV-IL-21. The results showed that the levels of IL-21, IFN-γ, and TNF-α in nude mice treated with hUCMSCs-LV-IL-21 were increased markedly in comparison with the control group, hUCMSCs group and hUCMSCs-LV-Vec group, respectively as are shown in Figure 3C-E. Furthermore, we further identified whether IL-21 was expressed in ovarian cancer tissues in the mice. Figure 3F indicates that there was IL-21 protein expression in the tumor tissues, which may suggest that hUCMSCs based IL-21 participated in the therapy of ovarian cancer. Given the crucial role of NK cell activity in the immunosurveillance of tumors, which are related with the expression levels of NKG2D and MIC A molecules, we thus detected the molecular transcript expression of NKG2D and MIC A. Figure 3G-H show the results of mRNA expression of NKG2D and MIC A tested by qRT-PCR in hUCMSCs-LV-IL-21 group, hUCMSCs-LV-Vec group, hUCMSCs group and control group, respectively, in the tumor tissues. The mRNA expression of NKG2D and MIC A were actually upregulated in the mice treated with hUCMSCs-LV-IL-21 group in contrast to the other groups. These data suggested that hUCMSCs-LV-IL-21 could markedly inhibited ovarian cancer growth in the mouse model, and the elevated cytokines above-mentioned may assist in hUCMSCs-LV-IL-21’s antitumor activity.

### Down-regulation of β-catenin and cyclin-D1 in tumor tissues

**Figure 4:**
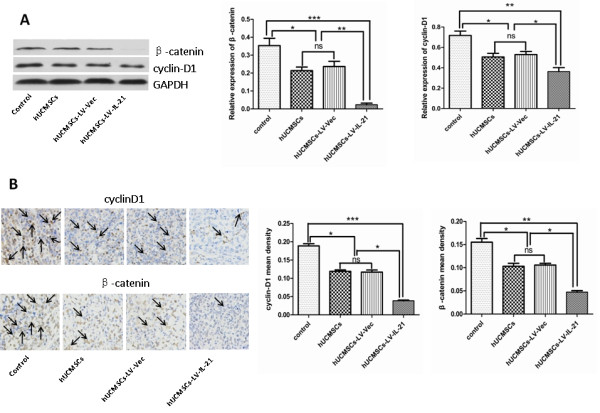
**The expression of β-catenin and cyclin-D1 in tumor tissues. A**. Western blotting results show the expression of β-catenin and cyclin-D1 in the tumor tissues; **B**. Immunohistochemistry also shows the expression of β-catenin and cyclin-D1. It was found that the expression of β-catenin and cyclin-D1 in tumor tissues from the mice treated with hUCMSCs-LV-IL-21 for four weeks was significantly decreased compared with the hUCMSCs group and hUCMSCs-LV-Vec group or markedly decreased compared with the control group. *p < 0.05, **p < 0.01, and ***p < 0.005. ns: no statistical significant.

It has been reported that hUCMSCs could inhibit tumor growth through the regulation of Wnt signal transduction pathway [34]. For this reason, we further investigated whether Wnt signal pathway was involved in the inhibitory effect on ovarian cancer mediated by hUCMSCs-LV-IL-21. The expression of β-catenin and cyclin-D1 was tested by immunohistochemistry and western blotting, respectively. As shown in Figure 4A, the results of western blotting showed that the expression of β-catenin and cyclin-D1 that is β-catenin target molecule was simultaneously down-regulated in tumor tissues of mice treated with hUCMSCs-LV-IL-21 in comparison with the control group, hUCMSCs group and hUCMSCs-LV-Vec group. Consistent with the observed reduction in the expression of β-catenin and cyclin-D1 detected by western blotting, the results of immunohistochemistry indicated the expression of β-catenin and cyclin-D1 in tumor tissues of mice treated with hUCMSCs-LV-IL-21 was also significantly decreased compared with the control group, hUCMSCs group and hUCMSCs-LV-Vec group, respectively (Figure 4B). The results implied that the secreted IL-21 from hUCMSCs-LV-IL-21 may assist in the inhibition of ovarian cancer growth in the mouse model by down-regulation of β-catenin and cyclin-D1 expression in Wnt/β-catenin signal transduction pathway.

### Histological analysis of the spleen, liver, lung, stomach and tumor tissues in the nude mice

**Figure 5:**
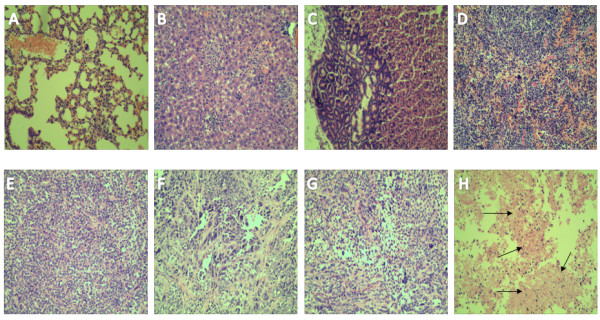
**Histopathology of normal tissues and tumor tissues in the nude mice (H&E 100×). A**-**D**. Absence of tumor formation in nude mice injected with 1 × 10^6^ hUCMSCs for 60 days. The tissue sections of lung **(A)**, liver **(B)**, stomach **(C)** and spleen **(D)** show no presence of any tumor cells; **E**-**H**. The tumor tissue histopathological sections were made in mice of the control group **(E)**, the hUCMSCs group **(F)**, the hUCMSCs-LV-Vec group **(G)**, and the hUCMSCs-LV- IL-21 group **(H)**, respectively. Section **E** shows the active growth of the tumor cells and obvious nucleic divisions. A few of the apoptosis tumor cells were observed in the tumor tissues of the mice treated with hUCMSCs **(F)** and hUCMSCs-LV-Vec **(G)**, but a little more necrotic, apoptotic tumor cells were found in Section **H**, which was collected from the tumor tissues from the mice treated with the hUCMSCs-LV-IL-21 group.

All the mice were killed 60 days after injection of 1 × 10^6^ hUCMSCs. In the 60-day observation, five nude mice appeared healthy with no clinical symptoms. No visible tumors were found around the transplantation sites or in any other regions of mice. Histological analysis also failed to detect any evidence of tumors in tissues such as lung, liver, stomach, and spleen (Figure 5A-D). These results suggested that the hUCMSCs did not form tumors in mice and thus could be potentially usable for hUCMSCs-based studies. In addition, histopathological analysis was also performed on the tumor tissues in the nude mice at 35 days. Figure 5E-H show that some necrotic, apoptotic tumor cells or vascular bleeding (H, arrows) may be attributable to increased tumoricidal activities of tumor-infiltrating immunocytes. In contrast, the active growth of the tumor cells and obvious nucleic divisions or diverse nucleic types were found in the mice challenged with the SKOV3 cells (E). It was also found that a few of the tumor-infiltrating immunocytes and a part of the apoptosis tumor cells were noticeable in the tumor tissues of the mice treated with hUCMSCs (F) and hUCMSCs-LV-Vec (G). The results of histological analysis suggested that the hUCMSCs-LV-IL-21 could induce nude mice to generate the immune responses to the ovarian cancer SKOV3 cells.

## Discussion

In the study, we first constructed the recombinant lentiviral vector for lasting the expression of IL-21, and then the hUCMSCs transducted by IL-21 gene packaged lentivirus had been injected into mouse ovarian cancer model to assess the effect of hUCMSCs-LV-IL-21 on ovarian cancer in vivo.

Our experimental results showed that hUCMSCs-LV-IL-21 induced powerful tumoricidal activities against human ovarian tumor in the xenograft-bearing Balb/c nude mouse model after the injection of SKOV3 cells. This effectiveness was reflected in the tumors showing slower growth and the smaller sizes (Figure 3), in the tumor cell necrosis, and in the observation that the vascular bleeding was more obvious in the tumor tissues of mice treated with hUCMSCs-LV-IL-21 group than other groups (Figure 5H).

The IL-21 secreted by the hUCMSCs-LV-IL-21 may stimulate the innate immune response to SKOV3 ovarian cells as well as induce hUCMSCs-LV-IL-21 to differentiate more immune cells, enhance the immune activity, and play a key role in killing tumor cells or drive tumor cell apoptosis. Although hUCMSCs can also elicit antitumor immune responses in contrast to the control group, this antitumor efficacy was especially true of the mice transplanted with hUCMSCs-LV-IL-21 that secrete IL-21 for long time.

To understand the mechanisms of antitumor efficacy induced by hUCMSCs- LV-IL-21, we examined the serum cytokines of IL-21, IFN-γ, and TNF-α, which have an important functions in antitumor immunity and their levels indirectly represent the capacity of rejection of tumor in bearing-tumor nude mice. The secreted IL-21 by hUCMSCs-LV-IL-21 can induce secondary cytokine production. The secreted IFN-γ and TNF-α by the NK cells or the macrophage, particularly the IFN-γ, can react to the NK cells, increase its cytotoxic activity, and play a key role in killing tumor cells or induce tumor cell apoptosis. The presented data showed that the IL-21, IFN-γ and TNF-α were respectively enhanced in the mice treated with hUCMSCs-LV-IL-21 group compared with other groups. Although it has been shown that serum levels of cytokines delivered by engineered stem cells have no obvious role in contrast to cytokine given systemically, the local production of cytokine appears to be the key for tumor growth attenuation. The stem cell-based gene therapy can also avoid potential systemic adverse effect of cytokines [35]. We speculate that hUCMSCs-LV-IL-21 elevated IFN-γ and TNF-α related to the secretion of IL-21, and that the elevated IFN-γ and TNF-α might markedly enhance the NK cytotoxicity that was supported by increase of expression of NKG2D and MIC A in the tumor tissues of the mice treated with hUCMSCs-LV-IL-21group. The enhanced NK cytotoxicity may be closely associated with the tumor cell necrosis, apoptosis, and the vascular bleeding in the tumor tissues (Figure 5H).

The canonical Wnt signaling pathway includes stabilization of cytosolic β-catenin, nuclear translocation, and gene regulation. Genes regulated by Wnt signaling are involved in metabolism, proliferation, cell cycle, and apoptosis [36]. Therefore, the proliferation of tumor cells can be influenced by regulating canonical Wnt signaling pathway [34,37]. It has been suggested that hUCMSCs could inhibit tumor growth through the regulation of this pathway [34]. We supposed that canonical Wnt signaling pathway might be involved in the inhibitory effects on ovarian cancer cells mediated by hUCMSCs-LV-IL-21. To test the hypothesis, we examined the molecular expression of β-catenin in tumor tissues. The results of western blotting and immunohistochemistry showed that the expression of β-catenin was down regulated in tumor tissues of mice treated with hUCMSCs, hUCMSCs-LV-Vec and hUCMSCs-LV-IL-21, respectively, especially in the hUCMSCs-LV-IL-21 group. To further evaluate whether known β-catenin target molecule was also reduced, we analyzed the expression of cyclin-D1. Consistent with the observed reduction in β-catenin, the cyclin-D1 expression was also down-regulated in same samples. We guess that the hUCMSCs-LV-IL-21 reduced β-catenin expression and might decrease β-catenin translocation to the nucleus to decrease the expression of cyclin-D1 that may reduce the transcription of its specific target gene, resulting in inhibition of tumor cell growth [34].

In conclusion, our data show that hUCMSCs display very similar characteristics to bone marrow-MSCs and could represent a valuable alternative for cell-based therapies. Transplanting hUCMSCs-LV-IL-21 into SKOV3 ovarian cancer xenograft-bearing nude mice has a definite therapeutic effect on inhibition of ovarian cancer growth. This augmented the therapeutic efficacy may contribute to the secreted IL-21 from hUCMSCs-LV-IL-21 and increase of the expression of NKG2D and MIC A, and assisting in exerting an antitumor effect through augmenting NK cytotoxicity. We speculate that the hUCMSCs-LV-IL-21 play an anti-tumor effect through regulating canonical Wnt signaling pathway. A more detailed understanding of the β-catenin and cyclin-D1 role in Wnt signaling pathway may be helpful in the investigation of the molecular mechanisms presented in hUCMSCs-LV-IL-21, and may improve therapeutic strategies involving hUCMSCs-LV-IL-21-mediated targeting therapy of ovarian cancer.

## Competing interests

The authors declare that they have no competing interests.

## Authors’ contributions

YZ, JW and MR carried out the experiments described in the manuscripts, developed the technique described in the manuscript, and participated in the writing of the manuscript. ML, DC, JC, FS, and XW participated in most of the experiments. JD contributed to the design of the experiments and to the writing of the manuscript. All authors have read and approved the final manuscript.
